# Metal–Support Interaction in Pt Nanodisk–Carbon Nitride Catalyst: Insight from Theory and Experiment

**DOI:** 10.3390/nano14110921

**Published:** 2024-05-24

**Authors:** Esmail Doustkhah, Ahmed Kotb, Timuçin Balkan, Mohammad Hussein Naseef Assadi

**Affiliations:** 1 Chemistry Department, Faculty of Engineering and Natural Sciences, Istinye University, Sarıyer, Istanbul 34396, Türkiye; h.assadi.2008@ieee.org; 2 Chemistry Department, Faculty of Science, Al-Azhar University, Assiut 71524, Egypt; 3 Chemistry Department, Koç University, Istanbul 34450, Türkiye; 4 Tüpraş Energy Center (KUTEM), Koç University, Istanbul 34450, Türkiye; 5 RIKEN Center for Emergent Matter Science (CEMS), Wako 351-0198, Japan

**Keywords:** metal–support interaction, hard template, crystalline Pt, oxygen reduction reaction (ORR), porous layered nitride, density functional theory, *d*-band theory

## Abstract

Metal–support interaction plays a critical role in determining the eventual catalytic activity of metals loaded on supporting substrates. This interaction can sometimes cause a significant drop in the metallic property of the loaded metal and, hence, a drop in catalytic activity in the reactions, especially in those for which low charge carrier transfer resistance is a necessary parameter. Therefore, there should be a case-by-case experimental or theoretical (or both) in-depth investigation to understand the role of support on each metal. Here, onto a layered porous carbon nitride (g-CN), we grew single crystalline Pt nanodisks (Pt@g-CN) with a lateral average size of 21 nm, followed by various characterisations such as electron microscopy techniques, and the measurement of electrocatalytic activity in the O_2_ reduction reaction (ORR). We found that intercalating Pt nanodisks in the g-CN interlayers causes an increase in electrocatalytic activity. We investigated the bonding mechanism between carbon support and platinum using density functional theory and applied the *d*-band theory to understand the catalytic performance. Analysis of Pt’s density of states and electronic population across layers sheds light on the catalytic behaviour of Pt nanoparticles, particularly in relation to their thickness and proximity to the g-CN support interface. Our simulation reveals an optimum thickness of ~11 Å, under which the catalytic performance deteriorates.

## 1. Introduction

Investigating the synthesis of atomically thin noble metals and their stabilisation can reveal new aspects of metals absent in bulk and isotropically synthesised nanoscaled metal structures [1,2,3]. However, the stabilisation of these metals is cumbersome due to the high cohesive energy that causes the metals to aggregate into larger sizes. So far, only a few methods have been available to synthesise such atomically thin materials, including templating approaches [4,5], the use of some specific precursors [1], and topotactic conversion of layered oxide into layered metal by reduction [2]. Still, in the case of the templating method, the final metal may undergo further aggregation if the template is removed from the final metal. In such a case, the metal’s interlayer can stack and eventually form a bulkier material. When the hard template remains with the metal nanosheets, a metal-supported hybrid nanostructure is formed, helping the metal retain its thin morphology. Here, the final material can be utilised as a stable candidate for various fields of catalysis [4]. In such a case, the hard template supports the metal. Therefore, the nature and features of support influence the metal through what has recently been distinguished as the metal–support interaction (MSI) [6,7]. The support–metal interface determines the electronic destiny of states at the catalyst reactant interface [8]. The MSI phenomenon in catalysis has been considered in terms of different aspects, such as leaching, stability, conductivity, and the electronic properties of metal impacted by the support [6,9]. Studying all these parameters all at once can be somehow overwhelming. In addition, the MSI effects vary from one metal or support to another. Therefore, a comprehensive case-by-case investigation should be performed for each metal loaded on a specific support. For instance, the electron population in the valence band, the band structure, density of electron state (DOS) [8], the ensemble of the supported metal [10], and the available surface area (active catalyst surface area) [11] can also be influenced by the type of support. The *d*-band theory is a promising approach to investigating the electronic properties of the supported metal [12]. From the *d*-band shifts, electronic changes in the supported metal can also be distinguished, explaining the changes in the catalytic activity. Accordingly, the *d*-band centre in metal nanostructures is responsible for the charge transfer and, therefore, the catalytic performance [13,14].

Platinum is one of the highly active metals in catalysis that has been used in many types of redox catalysis applications. Consequently, Pt has been the subject of many investigations in sizes engineered from nanoscale to a single atom [15]. However, the shape and types of support can still play a game-changing role in the catalytic activity of the supported platinum. Recently, Pt nanoparticles supported on TiO_2_ have been investigated through *d*-band theoretical calculations, showing that the *d*-band centre shifts from −2.2 to −2.3 eV because of the slight charge transfer across the Pt/TiO_2_ interface [13]. Likewise, Pt with different particle sizes on ceria (CeO_2_) was demonstrated to have particle-size-dependent charge transfer properties that can affect the catalytic impact [16]. We also previously demonstrated that Pd nanoparticles’ electronic state can be significantly influenced by the support’s surface and its functional groups, significantly altering the catalytic activity [8,17].

In this article, using carbon nitride material as a support, we demonstrate that Pt nanodisks with atomic thickness and significantly sizeable lateral dimensions can be synthesised as a single crystal particle. Through density functional theories, we also study this material’s charge distribution through Pt and its effect on its catalytic performance. We also investigate the loaded Pt nanostructures in the oxygen electroreduction reaction (ORR) and show how Pt nanodisks can influence the catalytic performance of carbon nitride in ORR.

## 2. Materials and Methods

### 2.1. Chemical and Apparatus

Layered sodium silicate was purchased from Nippon Chemical Industrial Co. Ltd. (Tokyo, Japan), Pt(acac)_2_ (97%) was purchased from Sigma-Aldrich (Darmstadt, Germany), and C_16_TMACl (95%) was purchased from FUJIFILM Wako Pure Chemical Corporation (Tokyo, Japan). Transmission electron microscopy (TEM) images were recorded on a JEOL (Tokyo, Japan) JEM-2100F microscope (operated at 300 kV).

### 2.2. Synthesis of g-CN and Pt@g-CN

The synthesis of g-CN was performed according to the literature published elsewhere [18]. After the synthesis of g-CN, for Pt growth in the interlayers, the obtained g-CN (3.5 mg) was dispersed in the DMF (20 mL) and sonicated with a probe sonicator for 2 h. Then, the Pt(acac)_2_ was added to the g-CN dispersion. After stirring the dispersion for 2 h, the precipitate was separated with centrifugation and washed with MeOH. Further, the obtained precipitate was redispersed in MeOH to reduce the adsorbed Pt complexes with NaBH_4_ (1 mM, 5 mL) at room temperature for 1 h. Finally, the product (Pt@g-CN) was obtained after centrifugation, washed with MeOH, and dried at 60 °C in a vacuum.

### 2.3. Electrocatalytic ORR

A homogenous ink was prepared as follows: Initially, the as-prepared samples were ground. Subsequently, 5.0 mg of the ground sample was dispersed in a mixture of 950 μL ethanol and water (volume ratio of 1:3), along with 50 μL of 5.0 wt% Nafion. After 60 min of sonication, a 5 μL suspension was deposited onto a glassy carbon electrode (GCE) with an area of 0.1256 cm^2^ and allowed to dry gradually. The resulting mass loading was 0.2 mg cm^−2^. Prior to the GCE activation, the electrode was pre-polished using 1 μm diamond and 0.05 μm alumina powder, followed by thorough washing with water. Electrochemical measurements were conducted using a CHI 842B instrument (Austin, TX, USA), including linear sweep voltammetry (LSV) at different rotator speeds at 10 mV s^−1^, and chronoamperometry (i-t) measurements in O_2_-saturated 0.1 M KOH at ≈ 0.5 V vs. the reversible hydrogen electrode (RHE) with a rotation speed of 1600 rpm. The electrolyte solution was purged with ultra-pure O_2_ and N_2_ for at least 60 min before ORR measurements. A platinum wire served as the counter electrode, while a silver–silver chloride (Ag/AgCl) electrode functioned as the reference electrode. The overall electron transfer numbers per oxygen molecule were determined from the slope of the Koutecky–Levich plots using the following equation:

(1) $$\frac{1}{J}=\frac{1}{J_{k}}+\frac{1}{J_{L}}=\frac{1}{J_{k}}+\left(\frac{1}{0.62nFC_{0}{\left(D_{0}\right)}^{2/3}\nu^{-1/6}}\times \frac{1}{\omega^{1/2}}\right),$$

where *J* denotes the current density, *J_k_* represents the kinetic current density, *J_L_* shows the diffusion-limited current density, *n* indicates the transferred electron number, *F* refers to the Faraday constant (*F* = 96,485 C mol^−1^), *C*_0_ represents bulk O_2_ concentration (1.2 × 10^−6^ mol cm^−3^), *D*_0_ is the O_2_ diffusion coefficient (1.9 × 10^−5^ cm^2^ s^−1^), *ν* is the electrolyte’s kinematic viscosity (0.01 cm^2^ s^−1^), and *ω* refers to the electrode rotating rate [19].

### 2.4. Computational Settings

Spin-polarised density functional theory (DFT) calculations were conducted using the VASP 5.2.2 package with projector-augmented wave potentials [20,21]. The dispersion effects were accounted for using the DFT-D3 level of theory as per the Grimme scheme [22,23]. The generalised gradient approximation was used to approximate the exchange-correlation functional [24]. The energy cut-off was set to 550 eV. The Brillouin Zone was sampled using a 3 × 3 × 1 Monkhorst–Pack mesh. The CN support was approximated with a cubic C_3_N_2_ structure from the literature [25] (Materials Project ID = mp-1188347). This g-CN structure has a cubic symmetry and a cell parameter of 5.085 Å, which is close enough to that of face-centred cubic Pt, which is 3.9239 Å. C_3_N_2_ structure was multiplied and cleaved along a lattice direction to form a 2*u* × 1*v* structure that was two-unit cells deep. On one side of the g-CN facet, 8 layers of cubic Pt were added to simulate the Pt@g-CN interface. The C atoms on the opposite g-CN facet were saturated with OH groups. Finally, a 20 Å vacuum slab was added to prevent the two ends of the interface structure from interacting with each other. The unit cell had a composition of C_48_N_32_(OH)_4_Pt_64_. This structure was carefully optimised to force components smaller than 0.01 eV/Å and with an energy threshold of 10^−7^ eV.

## 3. Results and Discussion

### 3.1. Synthesis and Characterisation of Pt@g-CN

**Figure 1:**
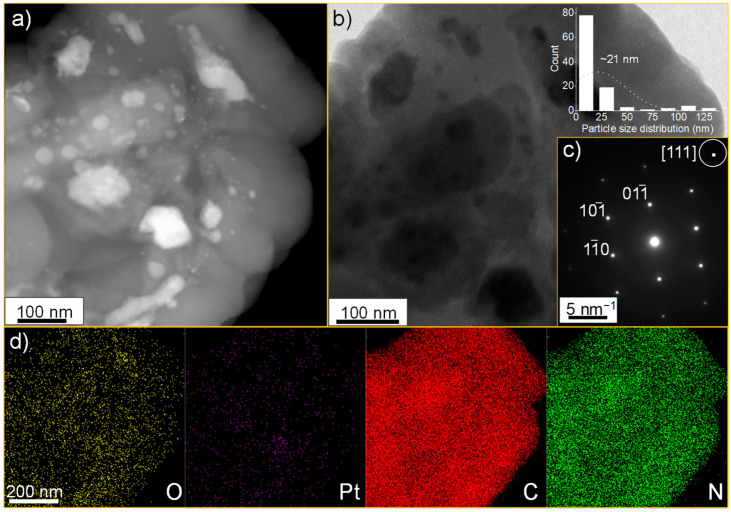
(**a**) HAADF-STEM image, (**b**) TEM image, (**c**) SAED pattern, and (**d**) HAADF-STEM-mapping images of Pt-grown g-CN.

In our previous investigation, we utilised layered silicate as a 2D nanospace for the synthesis of carbon nitride with a ratio of C_6_N (g-CN) and showed that this material is an excellent option for catalysis and supercapacitor applications. We also identified a thermodynamically stable configuration for C_6_N [18]. Here, we evaluated the capability of synthesised nanoporous g-CN as a template for the oriented growth of metallic Pt. We used a Pt(acac)_2_ organometallic precursor for the intercalation since this complex is hydrophobically favourable to interacting with the g-CN layers. The reduction of Pt ions was also conducted using sodium borohydride in a methanolic solution. In Figure 1a,b, HAADF-STEM and TEM images illustrate that the flakes of Pt with an average lateral size of ~21 nm are synthesised two-dimensionally in abundance, reaching a maximum dimension of 126 nm (Figure 1b, see inset). The SAED pattern also shows that the grown Pt nanodisks are single crystalline and are perpendicular to the [111] vector (Figure 1c). Furthermore, the HAADF-STEM mapping images also confirm the existence of Pt, O, N, and C elements (Figure 1d).

### 3.2. Theoretical Insight into Pt’s Performance from d-Band Theory

**Figure 2:**
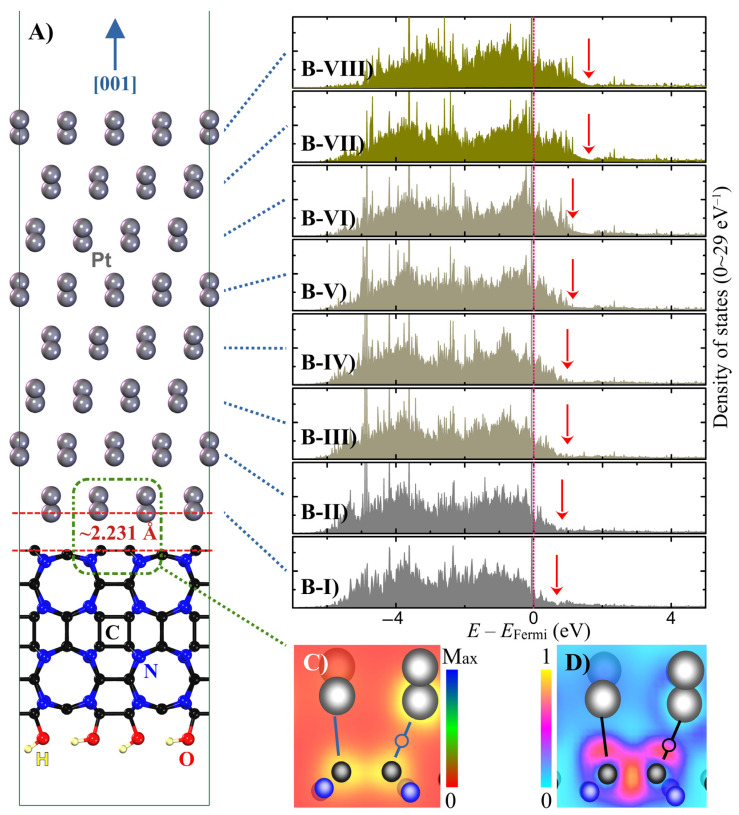
(**A**) Optimised Pt@g-CN structure; (**B-I**–**B-VIII**) the layer-resolved partial density of the Pt 5*d* states. The first layer is defined as the one directly interfacing with g-CN, while the eighth is the outermost layer catalytically interacting with the adsorbates; The red arrows indicate the extent of empty 5*d* states (**C**) charge density (*ρ*) map along the Pt-C bonds at Pt@g-CN the interface; (**D**) the corresponding electronic localisation function (*η*) map.

According to the optimised structure shown in Figure 2A, the distance between the g-CN support and Pt slab is only ~2.231 Å. This short distance promotes a strong chemical interaction between C and Pt, which may affect Pt’s electronic structure and, consequently, its catalytic potency. How the transition metal electronic structure determines its catalytic performance is generally understood through the *d*-band theory [26]. The *d*-band theory suggests that a transition metal catalyst’s ability to catalyse chemical reactions depends on the availability of empty (or partially filled) *d* orbitals on its facets. These empty *d* orbitals can interact with the molecular orbitals of reactant molecules, forming a new set of molecular orbitals that could facilitate the formation of new chemical bonds and lower the catalytic reaction’s activation energy. Here, two factors play more significant roles: the occupancy of the *d* orbitals and the *d*-band centre. As for occupancy, the extent to which these orbitals are filled or empty influences the catalytic activity. Surface atoms with only partially filled *d* orbitals are typically more active as catalysts. As for the *d*-band centre, a higher *d*-band centre within the valence band, or in other words, closer to the Fermi level, generally correlates with higher catalytic activity, as it indicates a greater ability of the transition metal to donate electrons to the catalytic products [27]. Both these factors can be investigated using the density of states of the relaxed structure of Figure 2A.

Figure 2B(I–VIII) shows the layer-resolved density of states (DOS) of the Pt ions. Here, Pt atoms were defined to belong to a specific layer if their *z* coordinates differed by less than 1 Å. We chose this criterion because, upon more nuanced inspection, we found that Pt atoms with this proximity had very similar DOS profiles. As a result, this definition of the layer would not result in any loss of accuracy while simplifying our analysis. Although the DOS plots offer a reliable visual tool to judge the Pt oxidation state and the location of 5*d* states, to objectively examine the variation in the Pt 5*d* characteristics across the layers, we integrated the 5*d* states in each layer to obtain the electronic population. The results of these integrations are presented in Table 1. Integration over the entire valence band shows the total occupied 5*d* electronic population. Integration over narrower ranges below the Fermi level indicates the portion of 5*d* electrons that is labile for catalysis, as those electrons are more accessible to interact with adsorbates and catalytic products. Because this range is not rigorously defined [8,28,29], here, we examine two ranges, the first from −3.5 eV to the Fermi level and the second from −3.0 to the Fermi level.

The eight subsets of Figure 2B(I–VIII) show that the Pt layers near the Pt@g-CN interface have a larger total 5*d* population compared to the upper layers far away from the interface. This trend is demonstrated by visibly smaller empty DOS above the Fermi level above for layer 1. For Pt atoms near the facet, the 5*d* population is remarkably smaller. This trend is marked by red arrows in Figure 2B(I–VIII). The DOS integration over the entire valence band shows that each Pt atom in layer 1 has 9.508 *e*, which is larger than the anticipated value of 9 *e* for Pt ([Xe] 4*f*^14^5*d*^9^6*s*^1^). Larger than 9 *d* electrons for Pt at the Pt@g-CN interface indicate a charge transfer from the g-CN support to the Pt layer right above the interface, reducing the amount of empty DOS in layer 1. Layers 2, 3, and 4 also show a 5*d* population larger than 9 *e* per Pt, although with decreasing significance. Contrary to the total 5*d* population throughout the entire valence band, the 5*d* population near the Fermi level, i.e., the catalytically most potent, consistently increases for the Pt layers that are away from the g-CN interface, reaching a maximum at layer 8, with a population of 5.847 *e*/Pt and 5.037 *e*/Pt when integrated from 3.5 eV and 3 eV below the Fermi level, respectively. One should note that in the absence of the g-CN support, the DOS and the electronic population at the top and bottom Pt layers would be the same due to symmetry. Accordingly, for the standalone Pt slab, we calculated a uniform population of 4.478 *e*/Pt and 3.487 *e*/Pt for integrations starting from −3.5 eV and −3 eV, respectively.

The density of states and the electronic population analyses show that the Pt nanoparticle thickness plays a critical role in determining its catalytic activity. For those Pt ions near the g-CN support interface, the charge transfer from C atoms into Pt’s 5*d* orbitals fills up the empty 5*d* states that were supposed to form antibonding molecular orbitals with the adsorbate molecules. Furthermore, the centre of the 5*d* states for those Pt ions closer to the g-CN interface shifts closer to the bottom of the valence band. Such a position inhibits their participation in catalysis. We only see a favourable electronic structure for Pt atoms in layer 5 and upwards. These results hint at minimum Pt nanoparticle thickness, comprising five atomic layers, for optimal catalytic performance when supported on g-CN. These five Pt atomic layers correspond to the thickness of ~11 Å.

Finally, we examine the bonding nature at the Pt@g-CN interface to elucidate the mechanism for charge transfer from C to Pt. The charge density profile (*ρ*) in the plane containing the Pt-C bond at the interface, shown in Figure 2C, indicates a diminishing charge density in the middle of the bond (marked with a circle). Such a *ρ* profile rules out the formation of any covalent or metallic bonds as these bonds tend to have a large charge density along the bonds [30,31]. Additionally, the electronic localisation function (*η*) profile shown in Figure 2D demonstrates an *η* peak near the C centre, which fades away at the mid-bond region. Note that *η* is a function derived from the calculated wave function that maps the probability density of electrons onto a scalar field, allowing for the visualisation of electron localisation [32]. Low charge density *ρ* and low probability of electronic localisation *η* in the mid-bond region strongly hint at a bonding with ionic character between C and Pt [33]. Such bonding is speculated to be weaker than the bond formed between Pd and oxide supports, which is more covalent [8].

### 3.3. Electrocatalytic Activity Investigations

**Figure 3:**
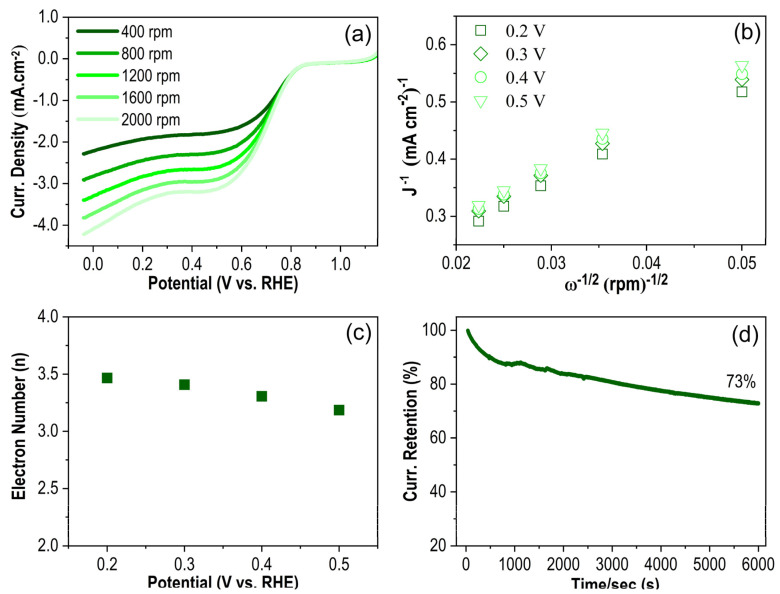
(**a**) RDE polarisation curves at different rotation rates at a scan rate of 10 mV/s; (**b**) Koutecky–Levich plots for the ORR at different electrode potentials; (**c**) calculated electron numbers at different potentials; (**d**) stability measurement of Pt@g-CN in O_2_ saturated 0.1 M KOH at ≈0.5 V.

**Figure 4:**
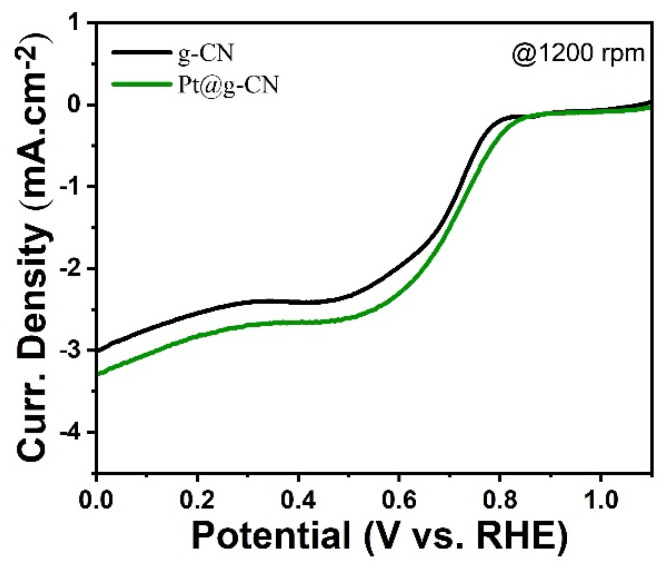
Comparison of RDE curves belonging to g-CN and Pt@g-CN at 1200 rpm.

Electrochemical measurements were carried out in an O_2_-purged KOH (0.1 M) solution to explore the catalytical activity of Pt@g-CN towards ORR. Figure 3a shows rotating disc electrode (RDE) polarisation curves at different rotational rates (from 400 up to 2000 rpm). As expected, as the rotation rate increases, the limiting current density increases since the diffusion layer becomes shorter. Additionally, a reduction peak originating from the ORR reaction is observed, which has an *E*_onset_ potential at ≈0.84 V vs. RHE. Compared to pristine g-CN (*E*_onset_ ≈ 0.78 V), this value is enhanced by almost 60 mV (Figure 4). Compared to the bulk (Bare) Pt, Pt@g-CN has a slightly lower *E*_onset_, which is expected because a pure Pt may have higher electrocatalytic activity, but the electrode preparation process is not economical [34,35,36]. The *E*_onset_ of the commercial Pt/C (≈1 V) is also marginally better than that of our proposed electrode. However, the conventional Pt/C electrodes also have a high Pt load compared to Pt@g-CN [37,38,39,40], increasing the cost significantly.

Electron transfer numbers during the ORR were determined via the Koutecky–Levich equation (see equation above) extracted from the RDE curves. Figure 3b shows a linear relationship between *J*^−1^ and *ω*^−1/2^ at different potentials that proposes a first-order ORR concerning dissolved O_2_ concentration. Figure 3c shows the calculated transferred electron numbers using the slope derived from Figure 3b. They were found to be between 3.2 and 3.5, which suggests a pseudo 4*e*^−^ reduction mechanism as opposed to the 2*e*^−^ pathway involving peroxide formation. It is expected from an ideal ORR catalyst to directly reduce O_2_ to OH^−^ by gaining 4*e*^−^ in a single step under alkaline conditions [41]. The stability measurement of the Pt@g-CN electrode was also analysed by chronoamperometry at 0.5 V vs. RHE in the O_2_-saturated KOH solution (Figure 3d). It is observed that the current value undesirably drops by 27% within almost 2 h. This drop might be due to the difference in the size of synthesised Pt nanoparticles (inset of Figure 1b), causing the Ostwald ripening phenomenon [42].

## 4. Conclusions

C_6_N carbon nitride was developed for the oriented growth of single-crystal Pt nanodisks (confirmed by SAED analysis). The charge transfer profile critically changes upon the metal–support interaction. In this regard, we performed density functional theory calculations at the DFT-D3 level of theory that includes dispersion effects. Our calculations demonstrate that the optimised Pt@g-CN structure exhibits a close ~2.231 Å between the g-CN support and Pt slab, resulting in an ionic interaction and influencing Pt’s catalytic potency, as explained by the *d*-band theory. The analysis of the density of states on a layer-by-layer basis reveals notable differences in the populations of Pt 5*d* electrons. Specifically, Pt layers close to the Pt@g-CN interface show higher electron populations. However, despite this, they exhibit lower catalytic activity. This is attributed to a shift in the *d*-band centre to lower energies within the valence band. These findings underscore the crucial influence of Pt nanoparticle thickness on achieving optimal catalytic performance. We also found that Pt nanodisks play a positive role in the catalytic improvement of g-CN in ORR, albeit not significantly, which is probably due to the atomically thin thickness of Pt, for which the ionicity at the interface with g-CN increases the electric resistivity.

## Figures and Tables

**Table 1:** The layer-resolved electronic population in the Pt 5*d* band presented as per Pt atom. Layer positions with respect to the Pt@g-CN interface are as in Figure 2. The last two columns show the electronic populations within narrower energy ranges below the Fermi level for which the 5*d* electrons are catalytically more labile.

Layer	Entire Valence Band	−3.5 ≤ *E*_Fermi_ ≤ 0	−3.0 ≤ *E*_Fermi_ ≤ 0
8 (Outermost)	8.927	5.847	5.037
7	8.961	5.124	4.396
6	8.992	5.227	4.455
5	8.988	5.235	4.527
4	9.343	5.170	4.528
3	9.548	5.202	4.533
2	9.605	5.218	4.543
1 (Pt@g-CN Interface)	9.580	5.387	4.665

## Data Availability

The experimental data supporting this study’s findings are available from the corresponding author, E.D., upon reasonable request. The theoretical data can be fully reproduced using the input parameters reported in Section 2.4.

## References

[1] Ando S., Yamamoto E., Kobayashi M., Kumatani A., Osada M. (2024). Facile Synthesis of Pd Nanosheets and Implications for Superior Catalytic Activity. ACS Nano.

[2] Takimoto D., Toma S., Suda Y., Shirokura T., Tokura Y., Fukuda K., Matsumoto M., Imai H., Sugimoto W. (2023). Platinum nanosheets synthesized via topotactic reduction of single-layer platinum oxide nanosheets for electrocatalysis. Nat. Commun..

[3] Ide Y., Matsuoka M., Ogawa M. (2010). Efficient Visible-Light-Induced Photocatalytic Activity on Gold-Nanoparticle-Supported Layered Titanate. J. Am. Chem. Soc..

[4] Doustkhah E., Rostamnia S., Tsunoji N., Henzie J., Takei T., Yamauchi Y., Ide Y. (2018). Templated synthesis of atomically-thin Ag nanocrystal catalysts in the interstitial space of a layered silicate. Chem. Commun..

[5] Wang W., Zhao Y., Ding Y. (2015). 2D ultrathin core–shell Pd@Ptmonolayer nanosheets: Defect-mediated thin film growth and enhanced oxygen reduction performance. Nanoscale.

[6] Farmer J.A., Campbell C.T. (2010). Ceria Maintains Smaller Metal Catalyst Particles by Strong Metal-Support Bonding. Science.

[7] Hu S., Li W.-X. (2021). Sabatier principle of metal-support interaction for design of ultrastable metal nanocatalysts. Science.

[8] Doustkhah E., Tsunoji N., Assadi M.H.N., Ide Y. (2023). Pd Thickness Optimization on Silicate Sheets for Improving Catalytic Activity. Adv. Mater. Interfaces.

[9] Lou Y., Xu J., Zhang Y., Pan C., Dong Y., Zhu Y. (2020). Metal-support interaction for heterogeneous catalysis: From nanoparticles to single atoms. Mater. Today Nano.

[10] Lee J.H., Cho J., Jeon M., Ridwan M., Park H.S., Choi S.H., Nam S.W., Han J., Lim T.-H., Ham H.C. (2016). Experimental and computational studies of formic acid dehydrogenation over PdAu: Influence of ensemble and ligand effects on catalysis. J. Mater. Chem. A.

[11] Bai J., Ke S., Song J., Wang K., Sun C., Zhang J., Dou M. (2022). Surface Engineering of Carbon-Supported Platinum as a Route to Electrocatalysts with Superior Durability and Activity for PEMFC Cathodes. ACS Appl. Mater. Interfaces.

[12] Ando F., Gunji T., Tanabe T., Fukano I., Abruña H.D., Wu J., Ohsaka T., Matsumoto F. (2021). Enhancement of the Oxygen Reduction Reaction Activity of Pt by Tuning Its d-Band Center via Transition Metal Oxide Support Interactions. ACS Catal..

[13] Aso R., Hojo H., Takahashi Y., Akashi T., Midoh Y., Ichihashi F., Nakajima H., Tamaoka T., Yubuta K., Nakanishi H. (2022). Direct identification of the charge state in a single platinum nanoparticle on titanium oxide. Science.

[14] Mohajer S., Sharif M.A., Aghdam A.H., Borjkhani M., Assadi M.H.N. (2023). Amplified hybrid surface plasmon polaritons in partially reduced graphene oxide supported on gold. Appl. Surf. Sci..

[15] Labinger J.A. (2017). Platinum-Catalyzed C–H Functionalization. Chem. Rev..

[16] Lykhach Y., Kozlov S.M., Skála T., Tovt A., Stetsovych V., Tsud N., Dvořák F., Johánek V., Neitzel A., Mysliveček J. (2016). Counting electrons on supported nanoparticles. Nat. Mater..

[17] Doustkhah E., Tsunoji N., Mine S., Toyao T., Shimizu K., Morooka T., Masuda T., Assadi M.H.N., Ide Y. (2024). Feeble Single-Atom Pd Catalysts for H_2_ Production from Formic Acid. ACS Appl. Mater. Interfaces.

[18] Doustkhah E., Kotb A., Tafazoli S., Balkan T., Kaya S., Hanaor D.A.H., Assadi M.H.N. (2023). Templated Synthesis of Exfoliated Porous Carbon with Dominant Graphitic Nitrogen. ACS Mater. Au.

[19] Zhang T., Wu J., Wang Z., Wei Z., Liu J., Gong X. (2022). Transfer of molecular oxygen and electrons improved by the regulation of C-N/C = O for highly efficient 2e-ORR. Chem. Eng. J..

[20] Kresse G., Furthmüller J. (1996). Efficiency of ab-initio total energy calculations for metals and semiconductors using a plane-wave basis set. Comput. Mater. Sci..

[21] Kresse G., Joubert D. (1999). From ultrasoft pseudopotentials to the projector augmented-wave method. Phys. Rev. B.

[22] Grimme S., Antony J., Ehrlich S., Krieg H. (2010). A consistent and accurate ab initio parametrization of density functional dispersion correction (DFT-D) for the 94 elements H-Pu. J. Chem. Phys..

[23] Grimme S., Ehrlich S., Goerigk L. (2011). Effect of the damping function in dispersion corrected density functional theory. J. Comput. Chem..

[24] Perdew J.P., Burke K., Ernzerhof M. (1996). Generalized Gradient Approximation Made Simple. Phys. Rev. Lett..

[25] Jain A., Ong S.P., Hautier G., Chen W., Richards W.D., Dacek S., Cholia S., Gunter D., Skinner D., Ceder G. (2013). Commentary: The Materials Project: A materials genome approach to accelerating materials innovation. APL Mater..

[26] Andersen M. (2023). Revelations of the d band. Nat. Catal..

[27] Zhao Z.-J., Liu S., Zha S., Cheng D., Studt F., Henkelman G., Gong J. (2019). Theory-guided design of catalytic materials using scaling relationships and reactivity descriptors. Nat. Rev. Mater..

[28] Seo D.-H., Lee J., Urban A., Malik R., Kang S., Ceder G. (2016). The structural and chemical origin of the oxygen redox activity in layered and cation-disordered Li-excess cathode materials. Nat. Chem..

[29] Assadi M.H.N., Fronzi M., Ford M., Shigeta Y. (2018). High-performance Na ion cathodes based on the ubiquitous and reversible O redox reaction. J. Mater. Chem. A.

[30] Savin A., Nesper R., Wengert S., Fässler T.F. (1997). ELF: The Electron Localization Function. Angew. Chem. Int. Ed..

[31] Pastor E., Sachs M., Selim S., Durrant J.R., Bakulin A.A., Walsh A. (2022). Electronic defects in metal oxide photocatalysts. Nat. Rev. Mater..

[32] Savin A., Silvi B., Colonna F. (1996). Topological analysis of the electron localization function applied to delocalized bonds. Can. J. Chem..

[33] Koumpouras K., Larsson J. (2020). Distinguishing between chemical bonding and physical binding using electron localization function (ELF). J. Phys. Condens. Matter.

[34] Yano H., Higuchi E., Uchida H., Watanabe M. (2006). Temperature Dependence of Oxygen Reduction Activity at Nafion-Coated Bulk Pt and Pt/Carbon Black Catalysts. J. Phys. Chem. B.

[35] Kariuki N.N., Myers D.J. (2021). Impact of Nickel Ions on the Oxygen Reduction Reaction Kinetics of Pt and on Oxygen Diffusion through Ionomer Thin Films. J. Electrochem. Soc..

[36] Li Y., Wu Q., Jiao S., Xu C., Wang L. (2013). Single Pt Nanowire Electrode: Preparation, Electrochemistry, and Electrocatalysis. Anal. Chem..

[37] Tu H., Zhang H., Song Y., Liu P., Hou Y., Xu B., Liao T., Guo J., Sun Z. (2023). Electronic Asymmetry Engineering of Fe–N–C Electrocatalyst via Adjacent Carbon Vacancy for Boosting Oxygen Reduction Reaction. Adv. Sci..

[38] Wang Y., Li Q., Zhang L., Wu Y., Chen H., Li T., Xu M., Bao S.-J. (2021). A gel-limiting strategy for large-scale fabrication of Fe–N–C single-atom ORR catalysts. J. Mater. Chem. A.

[39] Zhu Y., Zhang Z., Lei Z., Tan Y., Wu W., Mu S., Cheng N. (2020). Defect-enriched hollow porous Co–N-doped carbon for oxygen reduction reaction and Zn-Air batteries. Carbon.

[40] Sarkar S., Biswas A., Purkait T., Das M., Kamboj N., Dey R.S. (2020). Unravelling the Role of Fe–Mn Binary Active Sites Electrocatalyst for Efficient Oxygen Reduction Reaction and Rechargeable Zn-Air Batteries. Inorg. Chem..

[41] Balkan T., Küçükkeçeci H., Zarenezhad H., Kaya S., Metin Ö. (2020). One-pot synthesis of monodisperse copper–silver alloy nanoparticles and their composition-dependent electrocatalytic activity for oxygen reduction reaction. J. Alloys Compd..

[42] Borup R., Meyers J., Pivovar B., Kim Y.S., Mukundan R., Garland N., Myers D., Wilson M., Garzon F., Wood D. (2007). Scientific Aspects of Polymer Electrolyte Fuel Cell Durability and Degradation. Chem. Rev..

